# Factors associated with venous thromboembolic disease due to failed thromboprophylaxis

**DOI:** 10.1186/s12959-023-00566-4

**Published:** 2023-12-06

**Authors:** Santiago Grillo Pérez, Paula Ruiz-Talero, Oscar Mauricio Muñoz Velandia

**Affiliations:** 1 Internal Medicine Department, Faculty of Medicine, Pontificia Universidad Javeriana, Bogotá, Colombia; 2 Internal Medicine Department, Hospital Universitario San Ignacio, Bogotá, Colombia

**Keywords:** Thromboprophylaxis, Thromboprophylaxis failure, In-hospital Thrombosis, Pulmonary thromboembolism, Deep vein Thrombosis

## Abstract

**Introduction:**

Available evidence to identify factors independently associated with failed thromboprophylaxis (FT) in medical patients is insufficient. The present study seeks to evaluate in hospitalized patients, which clinical factors are associated with the development of FT.

**Materials and methods:**

A case-control study nested to a historical cohort, comparing patients who developed failed thromboprophylaxis (cases) with those who did not (controls). Univariate and multivariate regression analysis was performed to define the factors associated with FT.

**Results:**

We selected 204 cases and 408 controls (52.4% men, median age 63 years). Medical patients were 78.4%. The most frequent thromboprophylaxis scheme was enoxaparin. In the failed thromboprophylaxis group, most of the embolic events corresponded to pulmonary embolism (53.4%). Among cases, BMI was higher (26.3 vs. 25 kg/m2, p < 0.001), as was the proportion of patients with leukocytosis > 13,000 (27% vs. 18.9%, p:0.22), and patients who required intensive care management (48% vs. 24.8%, p < 0.001). Factors independently associated with FT were BMI (OR1.04;95%CI 1.00-1.09, p:0.39), active cancer (OR:1.63;95%IC 1.03–2.57, p:0.04), leukocytosis (OR:1.64;95%CI 1.05–2.57, p:0.03) and ICU requirement (OR:3.67;95%CI 2.31–5.83, p < 0.001).

**Conclusion:**

Our study suggests that the failed thromboprophylaxis is associated with high BMI, active cancer, leukocytosis, and ICU requirement. Future studies should evaluate whether there is benefit in adjusting the thromboprophylaxis scheme in patients with one or more of these factors.

## Introduction

Venous thromboembolic disease (VTD), defined as pulmonary embolism (PE) and deep vein thrombosis (DVT), is the third most common thromboembolic disease worldwide after acute myocardial infarction and stroke [1]. In the European Union, it causes more than 300,000 deaths [2] and is associated with an estimated cost of up to 8.5 billion euros [3]. It also generates high rates of disability in the short (27.1%) and medium term (7.1%) [4].

Risk factors for developing VTD include hospitalization [5, 6], which is why effective prevention strategies such as pharmacological thromboprophylaxis are used [7, 8]. However, up to 3% of patients on pharmacological thromboprophylaxis may experience thromboembolic events [9], a concept known as failed thromboprophylaxis (FT).

Various strategies have been proposed to reduce the risk of FT in surgical patients, such as increased doses of heparin in patients undergoing bariatric surgery [10, 11], or combined thromboprophylaxis (pharmacological and mechanical with pneumatic compression stockings) in patients at very high surgical risk, as proposed by the American Society of Hematology [12, 13]. At present, there is insufficient evidence to identify the factors independently associated with FT, and this information could help us to develop specific strategies to prevent FT in this population.

The present study investigates which factors are associated with the development of FT in a cohort of predominantly medical inpatients.

## Materials and methods

A case-control study, nested in a historical cohort, evaluated patients who received pharmacological thromboprophylaxis at the Hospital Universitario San Ignacio (HUSI) between 1 and 2019 and 31 December 2021, and compared those who presented a venous thromboembolic event despite pharmacological thromboprophylaxis (failed thromboprophylaxis) (cases) with those who did not (controls). Patients over 18 years old, hospitalized for more than 24 h and receiving pharmacological thromboprophylaxis with enoxaparin, unfractionated heparin or dalteparin during their hospital stay, were included. Patients with an indication for full anticoagulation prior to hospital admission were excluded. The study was approved by the institutional research and ethics committee with approval code 206/2021.

Patients were identified using two institutional databases: (1) an electronic registry, in which all patients receiving pharmacological thromboprophylaxis are systematically recorded (were case and controls were present), and (2) an institutional anticoagulation registry, which includes all patients diagnosed with PE and DVT (used to identify cases). Because of the low incidence of failed thromboprophylaxis and the large number of controls, case-control matching was considered on a 1:2 basis, adjusting for age (in ranges +/- 1 year), sex, type of pharmacological thromboprophylaxis, and date of hospitalization (+/- 90 days). When more than one control fulfilled the matching criteria, a randomization was used to select the control finally included in the analysis.

Once patients were identified, the institutional electronic medical records were reviewed for information on height, weight, presence of a central venous catheter, active cancer, history of venous thromboembolism, indication for hospitalization, surgery, missed doses, leukocytosis on admission, blood component transfusion, ICU stay and SARS COV 2 infection. The data were stored using a standardized format.

Cases were defined as patients without clinical suspicion of PE or symptomatic DVT on admission and in whom the diagnosis of acute pulmonary embolism was confirmed during hospitalization by chest angiography, ventilation-perfusion scan and/or confirmation of acute deep vein thrombosis by venous doppler of the lower limbs. Controls were patients with no clinical suspicion of PE or DVT on admission and in whom these were not documented during hospitalization.

Among the variables, pharmacological thromboprophylaxis was defined as treatment with enoxaparin at a dose of 40 mg subcutaneous per day, treatment with unfractionated heparin at a dose of 5000 U subcutaneous every 12 h, or dalteparin at a dose of 5000 U subcutaneous per day. Missed dose was defined as the non-application of pharmacological thromboprophylaxis despite its formulation according to thrombotic risk calculated by the Padua prediction score [14] in medical patients or the Caprini score for venous thromboembolism [15] in surgical patients, either because it was not prescribed by the attending physician or because it was not applied by the nursing staff.

For sample size calculation, we considered evaluating 10 cases for each variable of interest [16]. Considering that we identified 11 variables potentially associated with FT, a minimum required sample size of 110 cases and 220 controls was calculated. Categorical variables were reported as absolute numbers and percentages. Continuous variables were reported as median and interquartile range, as the assumption of normality was not met using the Shappiro-Wilk test. The t-test, Mann-Whitney U test or chi-squared test were used to compare cases and controls, depending on the type of variable. Finally, odds ratios (ORs) were calculated using a conditional logistic regression model with fixed effects, reporting first a univariate analysis and then a multivariate analysis, including in the analysis the variables that were significant in the univariate model or those reported to be associated with FT in previous studies. The selection of variables to be included in the final model was performed using the stepwise backward method. Those with a value of p < 0.05 were considered significant. The statistical package Stata 16 [17] was used for analysis. (Stata Statistical Software: Release 16. College Station, TX: Stata Corp LLC).

## Results

There were 1200 patients identified in the iinstitutional anticoagulation registry,, with newly diagnosed PE or DVT. Of this, 204 patients were identified to fulfill criteria for failed thromboprophylaxis (cases), accounting for 17% of all events. Of the 29,104 patients identified in the electronic thromboprophylaxis registry who received thromboprophylaxis and had no events (controls), we selected 408 patients matched by age, type of thromboprophylaxis, sex and time of hospitalization.

Most patients were male, with a median age of 63 years (IQR 51–74). Patients with a non-surgical indication for hospitalization were 78.4%. The most reported pharmacological thromboprophylaxis was enoxaparin (92%). 29% of patients in both groups had some form of active cancer.

The comparison between the case and control groups is shown in Table 1. There was higher incidence of hematologic cancer in the control group (33.88% vs. 16.64%), while prostate cancer was more frequent in the case group (4.13% vs. 15.25%) (p: 0.032). In the failed thromboprophylaxis group, most thrombotic events were PE (53.4%). Cases had a higher BMI (26.3 vs. 25 kg/m2, p < 0.001) and a higher proportion of patients with leukocytosis > 13,000 (27% vs. 18.9%, p:0.22). Cases also had more intensive care unit management (48% vs. 24.8%, p < 0.001) and were more frequently associated with SARS COV 2 infection (47.1% vs. 37.1%, p < 0.001). Longer hospitalization was observed in the case group (Median 19 days, IQR 12–30 vs. 13 days, IQR 7–21, p < 0.01), with a median of time since admission to VTE diagnosis in the case group of 8.5 days (IQR 5–15).

**Table 1:** Demographic and clinical characteristics of patients with failed and successful thromboprophylaxis

Variable	Failed thromboprophylaxis n = 204		Effective thromboprophylaxis n = 408	p value
Male, n (%)	107 (52.45)	214 (52.45)		1
Age in years, median (IQR)	63 (51–74)	63 (51–74)		1
Type of thromboprophylaxis, n (%)				
Dalteparin Enoxaparin Sodium heparin	2 (1.0) 188 (92.1) 14 (6.9)	4 (1.0) 376 (92.1) 28 (6.9)		1
BMI, kg/m^2^ median (IQR)	26.3 (23.6–30.1)	25.2 (22.2–29.0)		< 0.001
Permanent central venous catheter, n (%)	9 (4.4)	18 (4.4)		1
Active cancer, n (%)	59 (28.9)	121 (29.7)		0.851
History of VTE, n (%)	7 (3.4)	12 (2.9)		0.742
Indication for hospitalization, n (%)				
Medic Non-orthopedic surgical Orthopedic Surgical Neurosurgery Obstetrician	168 (82.3) 24 (11.8) 8 (3.9) 4 (2.0) 0 (0)	312 (76.5) 58 (14.2) 29 (7.1) 6 (1.5) 3 (0.7)		0.268
Surgery, n (%)*	38 (18.6)	91 (22.3)		0.293
Missed dose, n (%)				
0 1–2 3–4 > 5	140 (68.6) 46 (22.6) 8 (3.9) 10 (4.9)	297 (72.8) 74 (18.1) 27 (6.6) 10 (2.5)		0.116
Leukocytosis, n (%)	55 (27.0)	77 (18.9)		0.022
Transfusion of blood products, n (%)				
Packaged red blood cells Platelets Fresh frozen plasma Various	30 (14.7) 1 (0.5) 1 (0.5) 7 (3.4)	48 (11.8) 6 (1.5) 2 (0.5) 13 (3.2)		0.704
ICU requirement, n (%)	98 (48.0)	101 (24.8)		< 0.001
Infection with SARS COV 2, n (%)	96 (47.1)	135 (33.1)		< 0.001
Type of embolic event, n (%)				
PTE DVT PE y DVT	109 (53.4) 74 (36.3) 21 (10.3)	- - -		- - -

IQR, interquartile range; BMI, body mass index; VTE, venous thromboembolic disease; leukocytosis, > 13,000 leukocytes/mm^3^; ICU, intensive care unit; PTE, pulmonary thromboembolism; DVT, deep vein thrombosis

*The subtypes of surgery were also evaluated with no statistical difference found. These subcategories include: non-orthopedic, neurosurgical, orthopedic, obstetric, other

Table 2 shows the univariate and multivariate analysis of factors associated with failed thromboprophylaxis. In univariate analysis, FT was associated with BMI (OR 1.05; CI95% 1.01–1.09, p:0.011), presence of leukocytosis (OR 1.68; CI95% 1.23–2.53, p:0.012), ICU stay (OR 3.31; CI95% 2.23–4.90, p < 0.001) and SARS COV2 infection (OR 1.97; CI95% 1.36–2.87, p < 0.001). Multivariate analysis showed that factors independently associated with the development of FT were BMI (OR 1.04; CI95% 1.00-1.09, p: 0.039), presence of active cancer (OR 1.63; CI95% 1.03–2.57, p:0.036), leukocytosis (OR 1.64; CI95% 1.05–2.57, p:0.031) and ICU stay (OR 3.67; CI95% 2.31–5.83, p < 0.001). SARS COV2 infection was not significantly associated with FT on multivariate analysis (OR 1.29; CI95% 0.78–2.13, p: 0.312).

**Table 2:** Univariate and multivariate analysis of factors associated with failed thromboprophylaxis

Variable	Univariate analysis OR (95%CI) p value		Multivariate analysis OR (95%CI) p value	
BMI, kg/m^2^	1.05 (1.01–1.09)	0.011	1.04 (1.00-1.09)	0.039
Permanent central venous catheter	1.01 (0.44–2.31)	0.983	-	-
Active cancer	1.01 (0.68–1.48)	0.969	1.63 (1.03–2.57)	0.036
History of VTE	1.05 (0.39–2.85)	0.911	-	-
Indication for hospitalization i0				
Medic Non-orthopedic surgical Orthopedic Surgical Neurosurgery	Reference 0.77(0.46–1.30) 0.49 (0.21–1.14) 1.25 (0.35–4.49)	0.327 0.099 0.727	-	-
Surgery	0.82 (0.53–1.27)	0.371	-	-
Missed dose *	1.26 (0.88–1.81)	0.214	-	-
Leukocytosis	1.68 (1.23–2.53)	0.012	1.64 (1.05–2.57)	0.031
Transfusion of blood products	1.20 (0.78–1.84)	0.418	-	-
No transfusion Packaged red blood cells Platelets Fresh frozen plasma Various	Referencia 1.32 (0.81–2.16) 0.41 (0.05–3.43) 0.99 (0.09–10.97) 1.09 (0.43–2-79	0.269 0.410 0.996 0.859	- - - - -	- - - - -
ICU requirement	3.31 (2.23–4.90)	< 0.001	3.67 (2.31–5.83)	< 0.001
Infection with SARS COV 2	1.97 (1.36–2,87)	< 0.001	-	-

OR, Odds Ratio; CI, confidence interval; BMI, body mass index; VTE, venous thromboembolic disease; Leukocytosis, > 13,000 leukocytes/mm3; ICU, intensive care unit

*Missed dose, 0 missed dose vs. ≥ 1 missed dose calculation was performed

**Variables with p value > 0.05 in the univariate analysis were not included in the multivariate model

*** AIC of the model 496.46, Nagelkerke’s pseudo R2 0.085

## Discussion

In this study, we evaluated the clinical factors associated with failed thromboprophylaxis and found that BMI, active cancer, leukocytosis and need for intensive care unit were significantly associated with this outcome.

Obesity is known to be a prothrombotic state secondary to chronic inflammation [18] and is an independent factor that increases the risk of developing VTD by up to 6.2-fold [19]. Our results are consistent with those reported in the literature. To reduce the risk of FT in obese patients, strategies such as increasing the dose of low molecular weight heparin (LMWH) have been evaluated with inconsistent results. In a retrospective cohort study of 1335 patients, the incidence of VTD was similar in the high-dose and low-dose groups, with a higher incidence of bleeding complications in the high-dose group [20]. More recently, a meta-analysis of 6266 patients was published showing that the high-dose group had a lower incidence of VTD (OR: 0.47, 95% CI: 0.27–0.82, p:0.007) and a similar incidence of bleeding events (OR: 0.86, 95% CI: 0.69–1.08) compared with the standard-dose group [21]. Another proposed strategy for thromboprophylaxis in obese patients is to monitor anti-Xa levels and titrate the dose according to the results; however, this strategy also showed no benefit over the usual dose [22] and is therefore not currently recommended. At present, the appropriate dose of LMWH to reduce VTD in patients with a BMI > 30 kg/m2 remains controversial and is not currently recommended in international guidelines; however, this recommendation is likely to change in the future as additional evidence becomes available.

Our results are also consistent with the literature on active cancer as a factor associated with FT; it has been described that the malignant tumor expresses procoagulant proteins that, among other mechanisms, directly activate the coagulation cascade or platelets, thus representing a prothrombotic state [23]. In our study, the case group had a higher incidence of prostate cancer, whereas the control group had a higher incidence of hematologic cancer, however, prostate cancer is not typically associated with a higher incidence of thrombosis than other types of cancer [24], so it is unlikely it could change our results. Oncology patients have a 4.1-fold increased risk of thrombosis, rising to 6.5-fold in the setting of active chemotherapy [6], a risk that may increase during hospitalization. Therefore, the American Hematology Association guidelines for the prevention of thrombotic events consider combined thromboprophylaxis for surgery with high thrombotic risk and low bleeding risk [25]. A randomized clinical trial comparing weight-adjusted versus fixed-dose low-molecular-weight heparin in hospitalized patients found no difference in bleeding; however, the cumulative incidence of VTD at day 14 was 5.9% in the weight-adjusted arm (CI90%, 0-20.5%) [26]. The evidence provided by this study is very limited given the small sample size and lack of control for other potential cofounders such as obesity, so further studies are needed to make a recommendation in medical patients.

Leukocytosis was also associated with FT in our study. Leukocytosis can be caused by a variety of factors including neoplasia, drugs, hypersensitivity reactions and infection [27], with infection being the most common cause. Sepsis has been shown to be an independent risk factor for VTD (OR 1.74; 95%CI, 1.59–1.90). Risk factors associated with sepsis include age, diabetes, chronic kidney disease, chronic obstructive pulmonary disease, stroke, malignancy, and antibiotic use [28], most of which are risk factors for thrombosis. Among septic patients, those requiring mechanical ventilation [29] had a higher risk of thromboprophylaxis failure, suggesting that the greater the severity of the infectious process, the greater the risk of thrombosis.

ICU admission is recognized as a risk factor for failed thromboprophylaxis [30], which is consistent with our study. Risk factors have been identified in ICU patients such as mechanical ventilation (OR 1.56; 95% CI 0.23–10.45, p: 0.64), prolonged immobility (OR 2.14; 95% CI 0.11–40.87, p: 0.61) and femoral venous catheter use (OR 2.24, 95% CI 0.41–12.20, p: 0.35) [31]. In septic patients, ARDS has also been associated with an increased risk of thrombosis [32], all of which may be secondary to the prothrombotic state associated with these conditions. Pharmacological thromboprophylaxis in conjunction with mechanical thromboprophylaxis to reduce failed thromboprophylaxis is being studied and results are awaited [33, 34]; however, there are currently no studies to suggest additional interventions.

Strengths of this study include the high proportion of medical patients; however, the recommendations and findings of this study cannot be generalized to obstetric patients. Although this is an uncommon pathology, the required sample size was exceeded, which means greater statistical power. The results obtained are mostly in line with what has been demonstrated in the clinical literature. Limitations are associated with the case-control design in terms of control selection. We selected controls from a large cohort that included all patients who received thromboprophylaxis in our institution and matched them according to pre-defined criteria. However, residual confounding may be present due to unmeasured confounding variables. To overcome these limitations, large prospective cohorts will be required in the future. Additionally, considering the retrospective nature of our research, a causal relationship cannot be established.

## Conclusions

Our study suggests that BMI, active cancer, leukocytosis and need for intensive care are significantly and independently associated with thromboprophylaxis failure. The coexistence of these factors may suggest the use of alternative therapies to minimize this risk. Future studies should evaluate the potential benefits of these therapeutic options in medically managed patients.

## Data Availability

All of the material is owned by the authors.
